# Spatial compartmentalization of free radical formation and mitochondrial heterogeneity in bivalve gills revealed by live-imaging techniques

**DOI:** 10.1186/s12983-016-0137-1

**Published:** 2016-02-03

**Authors:** Georgina A. Rivera-Ingraham, Iara Rocchetta, Ulf Bickmeyer, Stefanie Meyer, Doris Abele

**Affiliations:** Alfred-Wegener-Institut Helmholtz-Zentrum für Polar- und Meeresforschung, Department of Biosciences, Am Handelshafen 12, 27570 Bremerhaven, Germany; Present address: UMR 9190 MARBEC, Groupe fonctionnel AEO, Bat. 24. CC092, Université de Montpellier, Place Eugène Bataillon, 34095 Montpellier, France; Present address: Laboratorio de Ecotoxicología Acuática, INIBIOMA, Consejo Nacional de Investigaciones Científicas y Técnicas (CONICET-COMAHUE), CEAN, Junín de los Andes, Neuquén, Argentina

**Keywords:** Bivalve, Gill, Live-imaging, Fluorescence, Mitochondria, ROS, RNS

## Abstract

**Background:**

Reactive oxygen (ROS) and nitrogen (RNS) species are produced during normal unstressed metabolic activity in aerobic tissues. Most analytical work uses tissue homogenates, and lacks spatial information on the tissue specific sites of actual ROS formation. Live-imaging techniques (LIT) utilize target-specific fluorescent dyes to visualize biochemical processes at cellular level.

**Results:**

Together with oxidative stress measurements, here we report application of LIT to bivalve gills for ex-vivo analysis of gill physiology and mapping of ROS and RNS formation in the living tissue. Our results indicate that a) mitochondria located in the basal parts of the epithelial cells close to the blood vessels are hyperpolarized with high Δψm, whereas b) the peripheral mitochondria close to the cilia have low (depolarized) Δψm. These mitochondria are densely packed (mitotracker Deep Red 633 staining), have acidic pH (Ageladine-A) and collocate with high formation of nitric oxide (DAF-2DA staining). NO formation is also observed in the endothelial cells surrounding the filament blood sinus. ROS (namely H_2_O_2_, HOO^•^ and ONOO^−^ radicals, assessed through C-H_2_DFFDA staining) are mainly formed within the blood sinus of the filaments and are likely to be produced by hemocytes as defense against invading pathogens. On the ventral bend of the gills, subepithelial mucus glands contain large mucous vacuoles showing higher fluorescence intensities for O_2_^•-^ than the rest of the tissue. Whether this O_2_^•-^ production is instrumental to mucus formation or serves antimicrobial protection of the gill surface is unknown. Cells of the ventral bends contain the superoxide forming mucocytes and show significantly higher protein carbonyl formation than the rest of the gill tissue.

**Conclusions:**

In summary, ROS and RNS formation is highly compartmentalized in bivalve gills under unstressed conditions. The main mechanisms are the differentiation of mitochondria membrane potential and basal ROS formation in inner and outer filament layers, as well as potentially antimicrobial ROS formation in the central blood vessel. Our results provide new insight into this subject and highlight the fact that studying ROS formation in tissue homogenates may not be adequate to understand the underlying mechanism in complex tissues.

## Background

Reactive oxygen and nitrogen species (ROS: H_2_O_2_, O_2_˙^−^, OH˙, HO_2_˙; RNS: NO˙, ONOO^−^) are by-products of cellular respiration in the mitochondria and of cellular detoxification mechanisms in the ER system. Both, ROS and RNS are strong redox agents, known to produce secondary radicals and oxidize cellular macromolecules such as membrane lipids, proteins, as well as nucleic acids (RNA and DNA). Both types of radicals are therefore considered as being cytotoxic and major drivers of cellular death and damage. The same radical species are also involved in cellular signaling including apoptotic death signals and further play an active role in processes such as muscle contraction [1], host-defense reactions (reviewed by [2–5]), and induction of mucus production via initiation of transcription factor cascades [6].

Under unstressed conditions ROS and RNS are produced in very low quantities, and bulk formation of ROS is usually measured in mitochondrial isolates [7, 8], cell preparations [9, 10], or even in tissue homogenates (e.g. [11, 12]). In vitro analysis of mitochondrial preparations or submitochondrial particles yields information on ROS forming mechanisms, albeit under non-physiological conditions, i.e. using artificial media, too high pO_2_, and too high respiratory substrate concentrations. On the other hand, using tissue homogenates or cell preparations, the ROS forming mechanisms and the subcellular structures of the ROS forming organelles (mitochondria, ER, vacuoles) remain unknown. The high reactivity of ROS and RNS implicates, however, that species specific lifetime and diffusion pathways inside cells and tissues are short. Thus, in living tissues steady state ROS concentrations are highly compartmentalized, and the distribution patterns of different ROS and RNS across sub-cellular structures can be the clue to their biochemical role and function. This compartmentalization can now be visualized using ROS/RNS sensitive probes, molecules which emit fluorescence upon reaction with the active species and after excitation at a distinct wavelength. Fluorescent probes enable minimal invasive detection of very small ROS/RNS quantities in living cells and tissues and facilitate the understanding of the dynamics and function of ROS production. For example, this type of studies may also help by providing input on the amount of ROS/RNS (in a qualitative manner) and thus setting the bases for further studies aiming to distinguish signaling or biosynthetic functions from toxic overproduction of ROS/RNS during an acute stress response.

In the past decades, live-imaging techniques have emerged as key tools for non-invasive studies of a wide variety of physiological parameters in- and ex-vivo in tissues and organs. In the field of oxidative stress, live-imaging techniques have been used to detect transient states of high ROS formation, and their use has enormously contributed to the understanding of small scale and high resolution spatial ROS dynamics in mammalian tissues (e.g. [13, 14]), while far less is known about marine invertebrate tissues.

Gills of marine and freshwater bivalves are multifunctional organs. They are the main respiratory tissues but, at the same time, filtering organs which collect food particles from the water column and transport them towards the mouth. Gill morphology and ultrastructure have been extensively studied, including the mechanisms underlying ciliary motility and beat frequency (e.g. [15–17]). The gills are also sensory organs for oxygen and salinity changes [18] and the first barrier against natural impacts (pathogens and natural toxins) or anthropogenic pollution. Most of the environmental stressors acting on marine animals produce oxidative stress and damage signals in the gills so that these organs are often targeted in environmental impact studies.

**Fig. 1:**
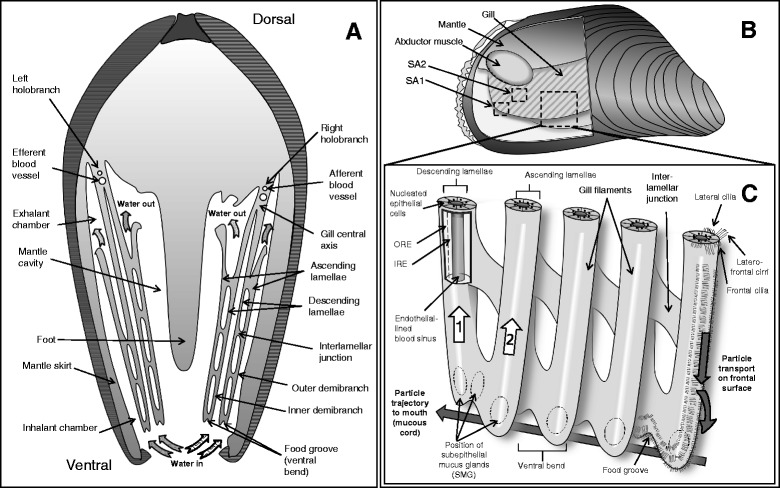
Schematic representations of the gill morphology of *M. edulis.* **a**: cross section (posterior view) of *M. edulis*. **b**: lateral view of a dissected *M. edulis* from the right side. Part of the right valve and mantle has been removed to reveal the right holobranch. **c**: detail view of the outer demibranch. SA1 and SA2 correspond to the different areas of the gill which were statistically compared in the study (ventral bend, and rest, respectively). ORE and IRE correspond to areas of the filament epithelial cells which were statistically compared in the study (outer regions and inner region, respectively). White arrow (1) indicates the direction of a slight augmentation in the number of mucocytes containing acid dominant mucopolysaccharids. White arrow (2) indicates the direction of a slight augmentation in the number of mucocytes containing neutral dominant mucopolysaccharids. For a detailed description on the histological distribution of SMGs, refer to [21]

The family Mytilidae has homorhabdic filibranch gills [19], one of the structurally most simple gill types, and has become a model organism for particle capture and transport (e.g. [20–23]). Briefly, homorhabdic (i.e. all filaments are of the same size) filibranch gills consist of long filaments, folded in a W-shape. Each gill (or holobranch) consists of two demibranchs (Fig. 1a) which join at a central axis. Each demibranch consists of two lamellae: descending lamellae face each other and drop from the central axis into the mantle cavity where they bend upwards to form the ascending lamellae. Lamellae are composed of numerous gill filaments joined through ciliary interlamellar junctions (Fig. 1a, b, c). The ventral bend of each demibranch forms a food groove, a longitudinal-oriented structure in which the food particle-mucus slurry is propelled towards the mouth by ventrally-directed water currents generated by ciliary beating (Fig. 1c). Gill filaments are densely packed with mucus forming mucocyte cells (around 13 to 33 per 100 μm) [21]. The dorsal part of the ventral bends contains a large number of subepithelial mucus glands (SMGs) [as described by 21] (Fig. 1c) which release mucus into the food groove to retain the collected food particles and transport them towards the labial palps and the mouth.

Here, we present a first spatially resolved analysis of ROS and RNS formation in different regions of the gill filaments of the common blue mussel *Mytilus edulis*, using multiphoton and confocal laser microscopy. We relate small scale patterns of ROS formation in unstressed bivalve gills to the regions of highest mitochondrial density and to the spatial pattern in mitochondrial activity state (membrane potential, Δψm). Non-invasive fluorescence imaging was applied to map different ROS/RNS in unstressed, intact sections of bivalve gill filaments to understand the dynamics and compartmentalization of basal ROS production in a complex tissue. Our aim was to better understand the multiple roles that ROS may have in gill physiology as deduced from their site of production, especially with respect to ciliary beat activity, blood vessel constriction, and antimicrobial protection of this multi-functional organ. Another interest was to investigate possible ROS involvement in mucus production within the SMGs.

## Results

### Gill micro-morphology and location of nuclei

**Fig. 2:**
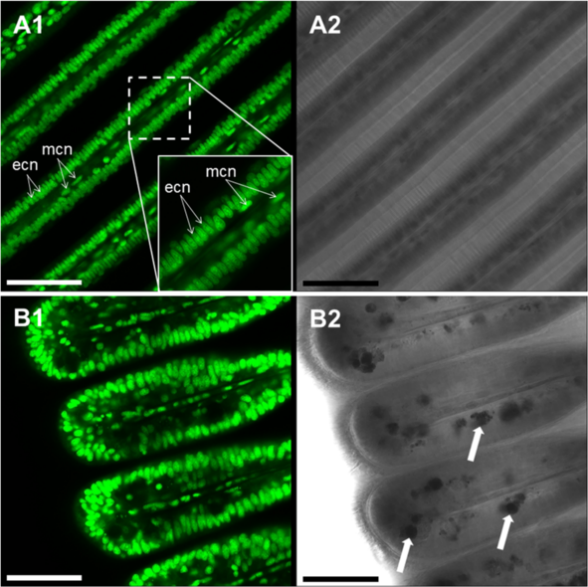
Representative Syto-13 staining in *M. edulis* gills (**a1**, **b1**) and corresponding transmission images (**a2**, **b2**). **a**: gill filaments with a capture showing a detailed view of nuclei structures. Filaments are composed of 1-layer epithelial cells with oval nuclei (ecn) perpendicular to the filament longitudinal axis. Note the muscle cells in the innermost areas of the filament with elongated nuclei (mcn) oriented parallel to the central blood sinus. **b**: Syto-13 staining of the ventral bend area of the gill with mucus-filled vacuoles (arrows). Scale bars = 50 μm

Staining with Syto-13 evidenced that the longitudinal sections of the gill filaments are composed of a 1-cell thick ciliated epithelial layer around a muscle cell coated blood sinus (Fig. 2). While the ciliated epithelial cells have large oval nuclei (18.9 ± 0.4 μm^2^) aligned as a palisade (see Fig. 2a1 insert), the long and thin endothelial (muscle) cells around the blood sinus are characterized by their elongated nuclei (Fig. 2).

SMGs located in the ventral bends of the gill filaments contain numerous large and irregular mucous-filled vacuoles with an average diameter of 57.6 ± 4.5 μm^2^. Vacuoles were more frequent in the filament sections adjacent to the mouth of each mussel.

### Mitochondrial density and membrane potentials

**Fig. 3:**
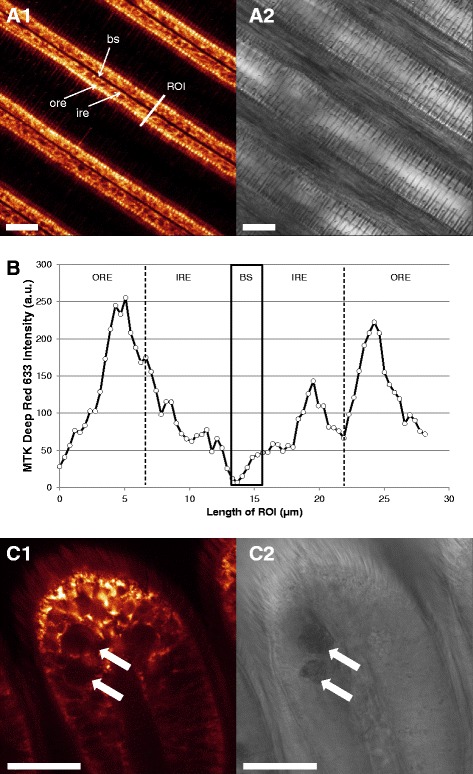
Representative Mitotracker Deep Red 633 staining of *M. edulis* gills. **a1**: overview of filamental structure (fluorescence image), **a2**: transmission image of the same area. **b**: fluorescence intensity profile across an average filament. Black box marks the limits of the blood sinus. Outer and inner region of epithelial cells were defined for statistical purposes as described in the method section. **c1**: view of ventral bend stained with MT Deep Red 633, **c2**: corresponding transmission image. Arrows mark mucus-filled vacuoles in SMGs. Bs = blood sinus, ore = outer region of the epithelial cells, ire = inner region of epithelial cells, ROI = region of interest. Scale bar = 25 μm

Staining with MTK Deep Red 633 (Fig. 3a) indicated the highest densities of mitochondria in the periphery of the gill filamental cells, directly beneath the cilia basal bodies (Fig. 3b). Statistical analysis revealed significant differences between the outer and the inner region of the epithelial cell longitudinal section, with the pattern of intensity: outer region > inner region > blood sinus (K = 28.45; *p* < 0.001). Vacuoles located within the SMGs were in no case stained by MTK Deep Red 633 (Fig. 3c).

**Fig. 4:**
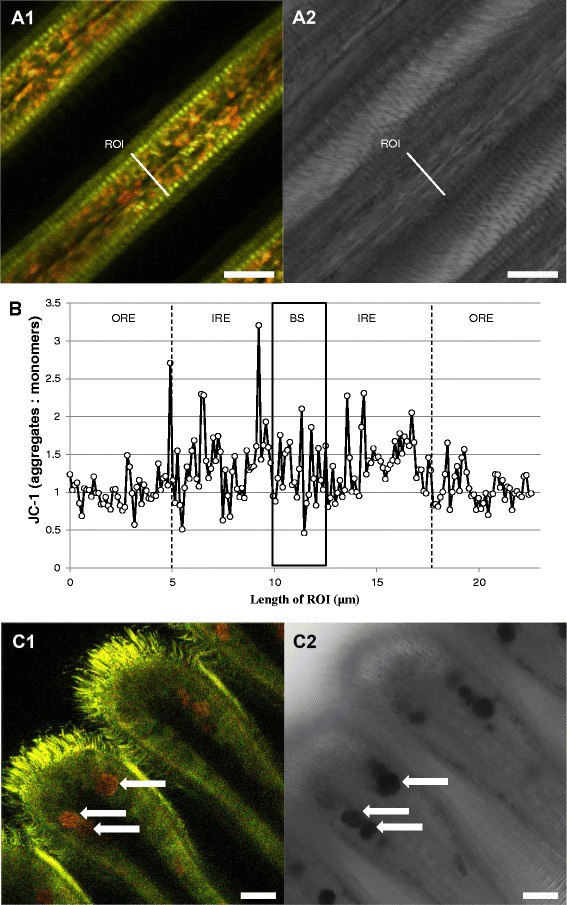
Representative JC-1 staining of gill filament visualizing areas with different mitochondrial membrane potential (Δψm). **a1**: overview of filament structure, **a2**: corresponding transmission image. **b**: fluorescence intensity ratio (aggregate/red: monomer/green) across a filament transect. Black box delimits the blood sinus. Outer and inner region of epithelial cells were defined for statistical purposes as described in the method section. **c1**: general view of the ventral bend, **c2**: corresponding transmission image. Arrows mark the mucus-filled vacuoles within the SMGs. ROI = region of interest. Scale bar = 20 μm

**Fig. 5:**
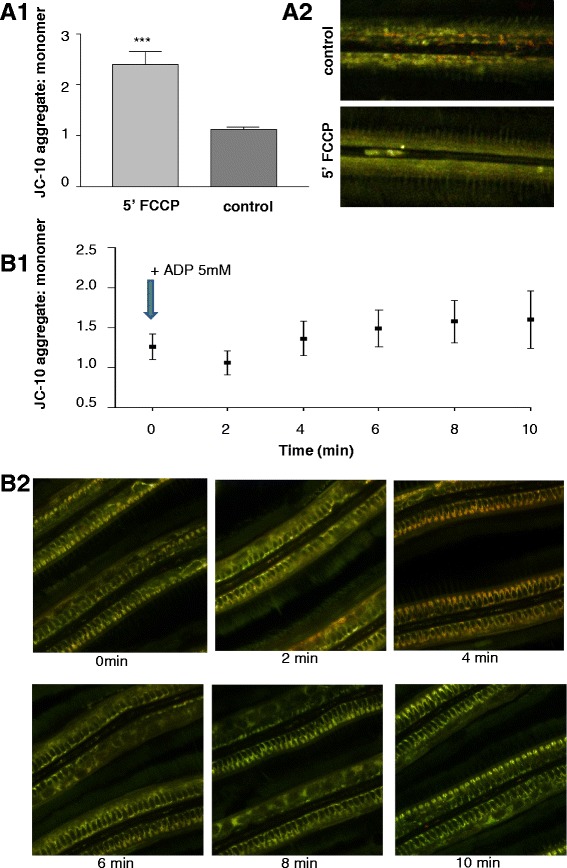
JC-1 / JC-10 control experiments. Effect of FCCP (**a**) (*** *p <* 0.001 (*t*-test) and ADP (**b**) (**p < 0.005, one way ANOVA) on JC-1 / JC-10 staining. Subfigures 1 show the average registered values while subfigures 2 show representative images of the experiments

JC-1 staining demonstrated two groups of mitochondria within the epithelial cells, clearly distinguishable according to membrane potential (Δψm), and presumably involved in distinct physiological processes. The outermost mitochondria directly below the cilia had the lowest (relatively depolarized) Δψm (JC-1 predominating in the green monomeric form indicating more active electron transport), whereas the rest of mitochondria had significantly higher membrane potential over the inner mitochondrial membrane (Fig. 4a). Statistical analyses confirmed significant differences of Δψm (aggregate: monomeric (outer region) 0.98 ± 0.02), (aggregate:monomeric (inner region) 1.20 ± 0.05) (K = 12.38; *p* < 0.001, Fig. 4b). Presence of both groups of mitochondria was confirmed by time laps experiments using JC-10 (Fig. 5). JC-1 and JC-10 staining both revealed depolarization of the mitochondrial membrane potential (green staining), following application of FCCP (Fig. 5a). This confirms the proper functioning of both dyes in *Mytilus* gill mitochondria. Further functional measurements (time laps) were performed using JC-10 and injecting ADP (end concentration 5 mM) directly into the microscopic chamber containing the gill filaments in measuring buffer. This application of ADP induced a short 2 min hyperpolarization in all experiments followed by depolarization (*N* = 6) of the membrane potential, indicating phosphorylation of ADP that caused the reduction of Δψm (Fig. 5b). The time laps experiments using JC-10 thus support a dynamic change of mitochondrial membrane potential in response to ADP availability to the gill cells. On the contrary, addition of ATP had no reproducible effect, presumable because any amount of ATP transported into the cells was directly consumed for ciliary beat activity.

In spite of being regarded as “mitochondria-specific”, JC-1 also strongly stained the mucus-containing vacuoles located within the SMGs. The aggregated form of JC-1 predominated in the SMGs (red fluorescence), and this may be indicating a high electrochemical potential across the membrane of the mucus filled vacuoles (Fig. 4c). However, to our knowledge this is the first report of JC-1 staining such structures.

### ROS/RNS distribution

**Fig. 6:**
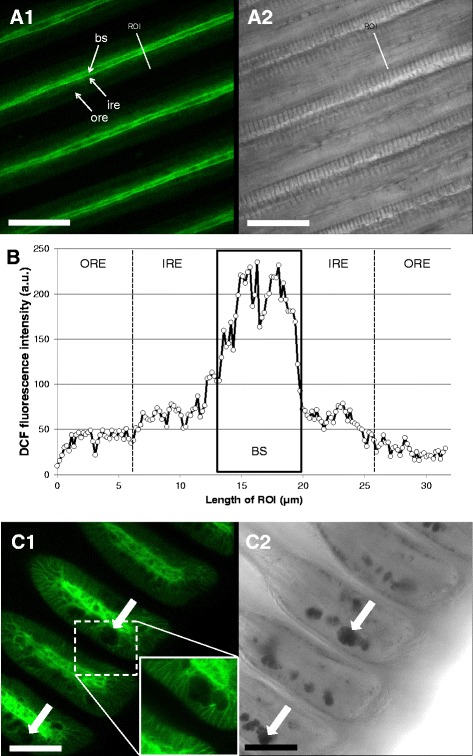
Representative DCF fluorescence patterns in *M. edulis* gills (ROS/RNS formation). **a1**: overview of filamental structure, **a2**: corresponding transmission image. **b**: DCF fluorescence intensity profile across a gill filament. Black box indicates the limits of blood sinus. Outer and inner region of epithelial cells were defined for statistical purposes as described in the method section. **c1**: view of the ventral bends with subepithelial mucus glands containing mucus vacuoles (white arrows) and corresponding transmission image (c2). Bs = blood sinus, ore = outer region of epithelial cells, ire = inner region of epithelial cells, ROI = region of interest. Scale bars = 50 μm

The distribution of maximal ROS and RNS formation differed greatly between different filament regions. We observed the maximum DCF fluorescence (C-H_2_DFFDA staining, ROS/RNS sensitive) within the blood sinus of the gill filaments (Fig. 6a), 1.8 fold higher (166.89 ± 7.41 a.u.) than the fluorescence recorded within the ciliated epithelial cells of the filament (47.00 ± 1.66 a.u.) (K = 92.86; *p* < 0.001) (Fig. 6b). Within these epithelial cells, significantly higher DCF fluorescence was registered in the innermost areas around the blood sinus (F = 216.28; *p* < 0.001). Mucus-filled vacuoles within SMGs were in no case stained by C-H_2_DFFDA (Fig. 6c).

**Fig. 7:**
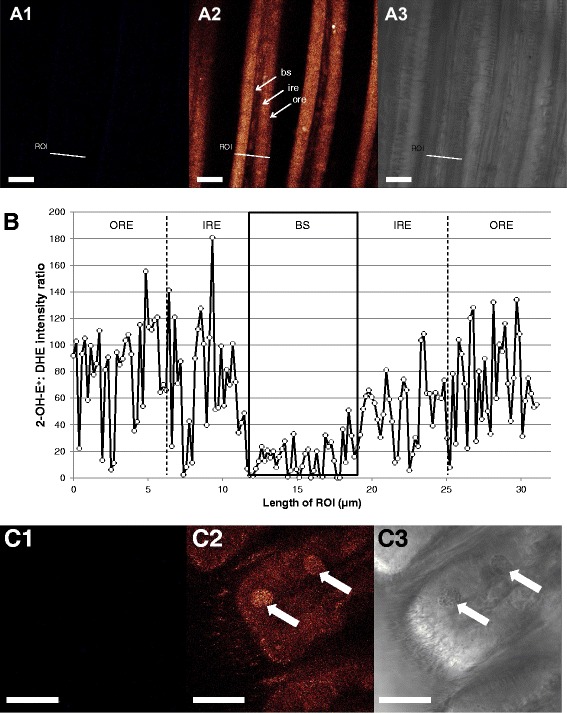
Representative 2-OH-E^+^ fluorescence patterns (O_2_^•-^ formation) in *M. edulis* gills. **a**: Overview of gill filaments, **a1**: blue-range fluorescence image (400–440 nm), **a2**: red-range fluorescence image (620–660 nm), **a3**: corresponding transmission image. **b**: fluorescence intensity profile across a filament. Black box indicates limits of blood sinus. Outer and inner region of epithelial cells were defined for statistical purposes as described in the method section. **c**: View of the ventral bend with subepithelial mucus glands containing mucus vacuoles (white arrows). **c1**: blue-range fluorescence image (400–440 nm), **c2**: red-range fluorescence image (620–660 nm), **c3**: corresponding transmission image. Bs = blood sinus, ore = outer region of epithelial cells, ire = inner region of epithelial cells, mc = mucocyte, ROI = region of interest. Scale bar = 25 μm

Contrary to the results obtained with C-H_2_DFFDA staining, the highest O_2_^•-^ concentrations (DHE staining) were observed within the epithelial cells (Fig. 7a). The 2-OH-E^+^:DHE fluorescence ratio in the epithelial cells of the filament (37.46 ± 3.34) was 2.4 fold higher than in the blood sinus (15.46 ± 2.02) (K = 55.47; *p* < 0.001) (Fig. 7b). Within epithelial cells, superoxide formation was higher in the outermost areas of the cells, as 2-OH-E^+^:DHE fluorescence ratio was significantly higher than in areas adjacent to the blood sinus (F = 6.906; *p* = 0.01). This staining also provided specific patterns in the ventral bend areas, where SMG vacuoles were intensively stained by DHE indicating higher concentration of superoxide to be present than in the other areas of the ventral bend cells (Fig. 7c). However, in neither of the cases (filaments or ventral bend areas) DHE staining provided structurally-resolved fluorescence patterns.

**Fig. 8:**
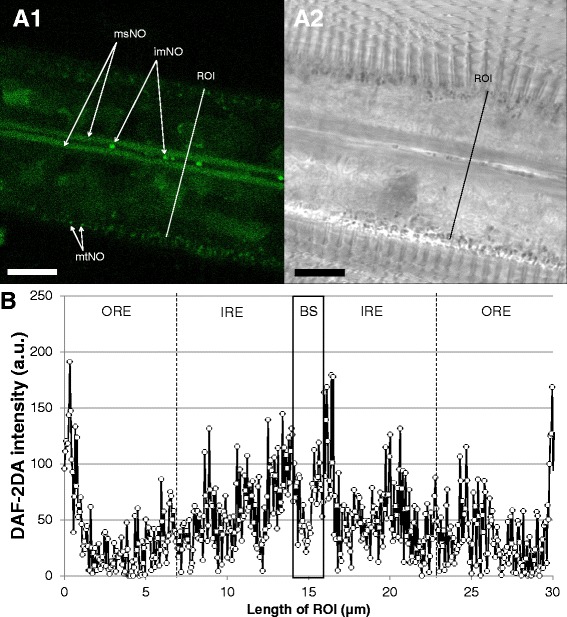
Representative DAF-2DA staining of *M. edulis* gill filaments (NO formation). **a1**: fluorescence (DAF-2T) image of filamental section, **a2** corresponding transmission image. **b**: fluorescence intensity profile across an average filament. Black box indicates the blood sinus inner area. imNO = immunocyte-associated NO formation, msNO = muscle cell-associated NO formation, mtNO = mitochondria-associated NO formation, Scale bar = 10 μm

DAF-2T fluorescence provides information regarding NO formation, which concentrated mainly in two regions of the gills filaments (Fig. 8a, b). Major DAF-2T fluorescence in the gills was recorded in the longitudinal cellular structures (muscle cells) surrounding the blood vessels and in the region of the blood vessel proximal mitochondria. DAF fluorescence in the peripheral region of densely packed cilia-associated mitochondria was less intense. Finally, small and likely circulating particles (<1 μm) within the blood vessels were strongly stained by DAF-2T. No significant DAF-2T fluorescence was recorded within the SMG vacuoles in the ventral bends of the gill filaments.

### pH measurements

**Fig. 9:**
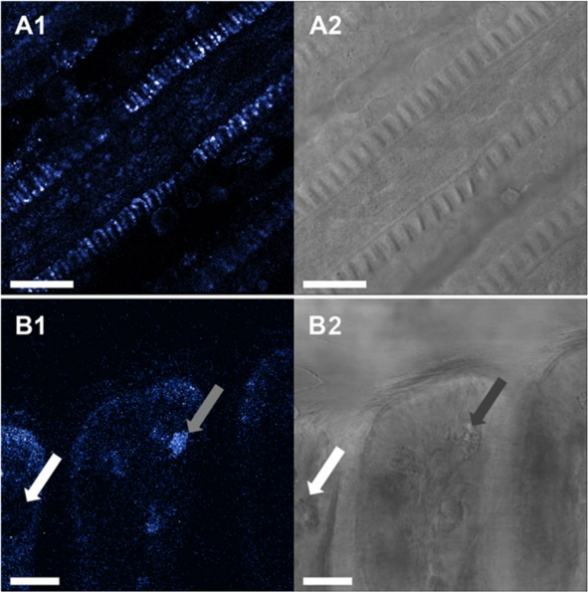
**a1**: representative ageladine-a pH staining of filament section, **a2**: corresponding transmission image. **b1**: representative ageladine-a pH staining of the ventral bend, **b2**: transmission image. pH varied among SMG mucus vesicles between acidic (grey arrow) and less acidic vesicles (white arrow). Scale bar = 20 μm

Ageladine-a pH-sensitive staining resolves a pH range from 4 to 8 [24] and evidenced that the areas of the gills with the lowest, i.e. most acidic, pH values were the areas of the cilia-associated mitochondria (Fig. 9a). Some mucus vacuoles had low pH values (Fig. 9b), but in most of the glands the pH did not differ from the rest of the tissue (presumably between 7.5 and 8). No other conspicuous differences in pH were observed in the gill filament structure.

### Antioxidant defense and oxidative damage

**Fig. 10:**
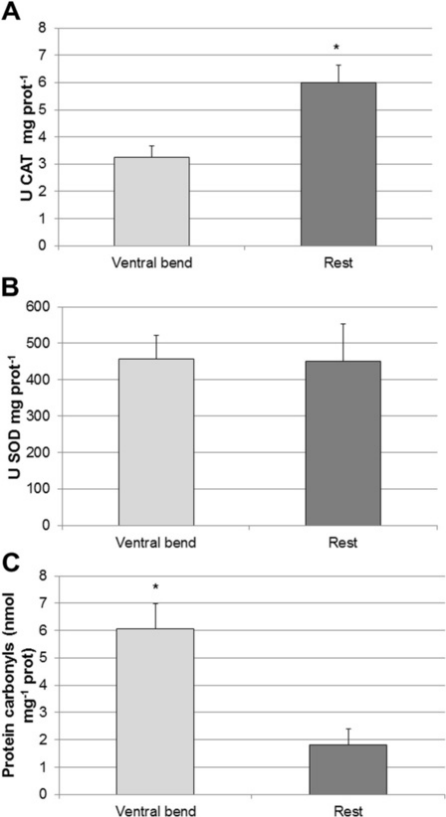
Antioxidant defense and oxidative damage measured in ventral bends and longitudinal sections of gill filaments as **a**) CAT activity, **b**) SOD activity and **c**) PC content. Values are expressed as mean ± SEM. * *p* < 0.05

Our analyses of antioxidant enzyme activities revealed differences between the outer ventral bend region and the rest of the gills: the ventral bends had significantly lower CAT activities than the rest of the gill tissue (F = 12.28; *p* < 0.05) (Fig. 10a), but no differences were observed in SOD activities (F = 0.004; *p =* n.s.) (Fig. 10b). Protein carbonyl (PC) content was higher in ventral bends (F = 16.53; *p* < 0.01), indicating that these areas are subject to higher oxidative stress than the rest of the gill tissue (Fig. 10c).

## Discussion

To our knowledge this study provides the first record of the mitochondrial spatial distribution and the basal production patterns of different ROS/RNS in bivalve gill tissues.

### Epithelial cells show differential mitochondrial populations based on membrane potential

**Fig. 11:**
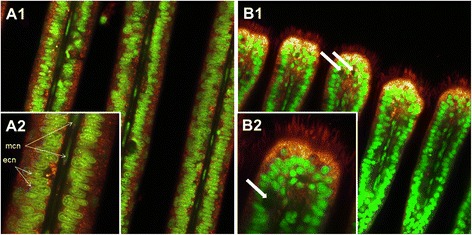
Representative Syto-13 (nuclei specific, green fluorescence) and Mitotracker Deep Red 633 (mitochondria specific, red fluorescence) combined staining. **a**.: General (**a1**) and detailed view (**a2**) of *M. edulis* central gill filament section. **b**.: General (**b1**) and detailed view (**b2**) of the ventral bend. ecn = epithelial-cell nuclei, mcn = muscle-cell nuclei. White arrows highlight the presence of mucus-filled vacuoles

Mitochondria are unequally distributed within the ciliated epithelial cells of the bivalve *Mytilus edulis*. They are mainly located in the peripheral region of gill epithelia cells, above the big basal nucleus and close to the cilia basal bodies, as confirmed by Syto-13 and MTK Deep Red 633 combined staining (Fig. 11). Across species and tissues, ciliated epithelia are commonly associated to basal layers of densely packed mitochondria (e.g. [25–27]), and the epithelial cells of *Mytilus* gills are a good example for this (e.g. [28, 29]). Kasuya & Miyoshi [30] found these mitochondria to be specifically abundant in the apical areas of well-developed ciliary rootlets of the *Mytilus* gills, and we also observed mitochondrial staining in ciliary rootlets. With JC-1 staining, the peripheral mitochondria appeared mostly green (more monomeric JC-1) indicating low membrane potential and active phosphorylation, presumably generating the ATP for ciliary beating.

To the contrary, many mitochondria in the central parts of the gills around the blood vessels were stained red (more aggregate with JC-1/10, indicative of hyperpolarized electrochemical membrane potential and reduced phosphorylation). Application of ADP at high concentration (5 mM) caused a short transient membrane depolarization as aggregate was lost from the mitochondrial matrix, and complete green staining was achieved when applying the uncoupler FCCP. Likewise FCCP abrogated transmembrane potential in mitochondria of isolated rat cardiac myocytes which led to a massive decrease of the aggregate (red) and a smaller increase of the monomer (green) JC-1/10 form in the uncoupled de-energized vertebrate mitochondria [31]. The increase of the green monomer, respectively the reduction of red aggregates, during prolonged experimental durations (>15 min) could be due to i) preferred bleaching of the red aggregate form or ii) wash-out of JC10 which was absent in the measuring buffer.

Mitochondrial populations with distinct Δψm were previously observed in different commercial cell cultures by Smiley et al. [32] and by Collins et al. [33], working with JC-1. Reers et al. [34] remarked that in the majority of cells so far investigated, mitochondria often have variable Δψm, frequently combining charged and non-charged mitochondria within cells [35].

Presence of idling mitochondria with high Δψm indicating low O_2_ consumption and low phosphorylation rates may be of advantage in respiratory epithelia. Theoretically, by limiting oxygen consumption in the gill epithelial cells, the “down-tuned” mitochondria around the blood vessel facilitate oxygen transport towards the hemolymph, thus serving the needs of the organism. Skulachev [35] proposed that a similar mechanism is involved for muscle fiber oxygen gradients in the periphery of capillary blood vessels in the sarcolemma. On the other hand, in the region containing the more highly polarized (red stained) mitochondria, we additionally recorded indications of NO formation (DAF-2DA staining). There are numerous literature reports of reversible depolarization of Δψm by NO (see review by [36]), which is also known to be a potent and reversible inhibitor of mitochondrial respiration in vertebrate cells. Such inhibition occurs in a variety of ways, but is mainly due to NO reversibly binding to the oxygen binding site of cytochrome oxidase, the terminal complex of the respiratory chain.

### Muscle-associated NO formation and NO forming cells in the hemolymph

The blood sinus is surrounded by longitudinal endothelial muscle cells with elongated nuclei as observed with Syto-13 staining (Fig. 2). In all analyzed samples, the highest DAF-2T fluorescence intensities were recorded within these longitudinal muscle cells (Fig. 8) or in their immediate vicinity (see above). We hypothesize that the presence of NO in the muscle cells is involved in muscle contraction/relaxation, causing a change in blood sinus diameter and regulating hemolymph flow in response to variable oxygen availability: wider blood vessels allowing for enhanced flow under hypoxic conditions. In vertebrates, NO mediates the activity of smooth (e.g. [37]) skeletal (e.g. [38, 39]) and cardiac muscles (e.g. [40]). Furthermore, NO has been reported to regulate hemolymph pressure in squid, *Sepia officinalis,* by acting as a vasodilator [41], and further experimental work is needed to investigate this putative function of NO in *M. edulis* gills.

We also observed significant NO concentrations within the blood sinus in most samples, linked to the presence of small vesicles (<1 μm) in the hemolymph (molluscan blood). This could be related to the antimicrobial defense system (review by [42]) of immunocytes which also produce NO directed against invading pathogens. NO has a cytotoxic effect which leads to bacterial clumping. NO formation by hemocyte cells has been reported from several bivalve species (see review by [43]), including *M. edulis* (e.g. [44]).

### ROS formation in the hemolymph is accompanied by enhanced catalase (CAT) defense

C-H_2_DFFDA staining also highlighted the intense ROS/RNS formation ongoing within the blood sinuses of gill filaments (Fig. 6). The same was observed in the context of our previous studies [45], where DCF fluorescence in hypoxically-incubated gills was 3.8-fold lower than in normoxically-treated gills, an indirect support that DCF fluorescence indeed measures ROS formation. The hemolymph of bivalves has previously been reported to contain significant amounts of H_2_O_2_. The bivalve *Astarte borealis* has an average hemolymph concentration of 10 μM H_2_O_2_ [46], which was measured by an alternative method using the fluorescence indicator scopoletin in a peroxidase catalyzed assay. This bivalve contains a giant composed and extracellular hemoglobin molecule, and the authors found evidence that H_2_O_2_ forms as a consequence of this hemoglobin autoxidizing to met-hemoglobin when exposing the bivalve to hypoxic treatment. In non-hemoglobin containing species the most plausible role of hemolymphatic H_2_O_2_ is, however, its well-known antimicrobial property. Hemocytes are known to be the main source of antimicrobial ROS formation in oxidative burst reactions reported for several bivalves (e.g. [47–50]) including *M. edulis* [51, 52]. However, we cannot discard the possibility that H_2_O_2_ could also be formed within the epithelial cells, through the activity of superoxide dismutase (SOD), and diffuses into the blood sinuses where we measured it. Nevertheless, oxidative damage to the epithelial and endothelial cells composing the gill filaments can be expected to result from these oxidative burst reactions. Our results suggest that ciliated epithelial cells contain a high CAT activity (as shown in Fig. 10a) which helps to prevent protein oxidation (Fig. 10c) and mitigates the oxidative stress effect that is associated to these oxidative burst reactions. A note of caution should, however, accompany our results and conclusions regarding the use of C-H_2_DFFDA (e.g. see review by [53]).

### Mucus-containing vacuoles are characterized by high O2•- formation: a double edge sword?

The mucus-containing vacuoles located within the SMGs (as observed in Fig. 2) appear to generate O_2_^•-^ radicals (Fig. 7), and our confocal analysis suggests superoxide to be the predominant radical in this cellular compartment. These superoxide anions may be secreted together with the mucus and have several functions. Gill tissues are not only respiratory epithelia, but also serve particle uptake through the secretion of mucus. However, a filtering tissue covered by mucus may additionally create a suitable habitat for microorganisms, which may have adverse effects for the respiratory surface. Superoxide anion is a relatively weak oxidant, but nevertheless has direct antimicrobial properties and may also be converted to the more persistent H_2_O_2_. Thus, producing a mucus with high concentrations of O_2_^•-^ could have a protective effect against the colonization of the gill surface by microbial tuffs. High concentrations of O_2_^•-^ have previously been detected in gill mucus of freshwater catfish [54]. Superoxide rich mucus is also used in many different cell types as a defense against pathogens. In fact, it is hypothesized that the properties of O_2_^•-^ as microbial killer is, at least in part, the basis for the function of phagocytic vacuoles, which are loaded with O_2_^•-^ resulting from burst activity of the NADPH oxidase (reviewed by [55]). Another good example is provided in the conjunctival tissue of the rabbit eye, which has been observed to secrete significant quantities of O_2_^•-^ into the mucus by granulocytes and another non-cellular ROS-generating oxidase system to protect the tissue from infections [56]. Yet another example are mammalian gastric mucosa cells, which are also known to produce O_2_^•-^ to regulate the defense response against bacterial growth [57]. But unlike the central areas of the gills, the ventral bends where these SMGs are located do not appear to counteract this superoxide production through enhanced SOD or CAT activities. In fact, SOD activity was similar to the rest of the tissue (Fig. 10b), which explains the higher oxidative damage (measured as PC content) in the ventral bends (Fig. 10c). Therefore, and unlike the rest of the gill filaments, cells located within the ventral bend areas of the gills are paying the price for the antibacterial protection and are subject to higher degrees of oxidative stress, leading to more active apoptotic turnover in bivalve gills compared to mantle or muscular tissue [58].

## Conclusions

We used non-invasive fluorescence imaging to demonstrate that gill filament epithelial cells are composed of two spatially segregated groups of mitochondria which differ in ΔΨm. We also mapped different ROS/RNS in different sections of bivalve gill filaments to understand the dynamics and compartmentalization of basal ROS production in intact and unstressed tissue. Interpretation of these spatial ROS/RNS patterns can be used to specify the functional role of these radical species within the gill structures. Released with the mucus, *M. edulis* may be using the antibacterial power of O_2_^•-^ to avoid the development of a biofilm on the gill surface and oxidize engulfed food particles. The high DCF fluorescence present in the hemolymph indicates defense against invading pathogens in the gill blood vessels. The free radical NO may have multiple functions with respect to cellular homeostasis in gill tissue. NO is produced in the internal region of the epithelial cells and may provide an additional mechanism for the polarized membrane potential in the mitochondria surrounding the blood vessels. Furthermore, the presence of significant NO concentrations around and within blood vessels suggest its involvement in muscle contraction and thus hemolymph pressure regulation, which may be of key importance when dealing with the low environmental oxygen concentrations that these bivalves can face in their benthic habitat. Finally, the presence of NO within the blood vessel may add to pathogen defense exerted by circulating immunocyte cells.

## Methods

### Animal collection and maintenance

Blue mussels *M. edulis* were collected at Sylt Island (Germany) (N 055° 01′ 323 E 008° 26′ 430) in December 2012. No permissions were required to conduct this task and it did not involve any protected species. Animals were transported to the Alfred-Wegener-Institut Helmholtz-Zentrum für Polar- und Meeresforschung (AWI) where they were maintained in aquaria at a constant temperature of 10 °C, 33 ‰ salinity and >99 % air saturation. Animals were cleaned from epibiotic growth and allowed to adapt to the aquarium conditions for 3 weeks in order to avoid any possible interference of the stress caused by the transport with the experimental results. Mussels were fed with live phytoplankton (Fa. Plankton Farm, Sycamore, USA) once per week, and 48 h were allowed between feeding and the start of the analysis to avoid possible interference of nutrition-induced increase in metabolic rates. Water circulation in the aquaria was stopped for four hours to allow filter feeding. Water quality was weekly assessed using Nanocolor® Tube Tests (Macherey-Nagel GmbH & Co. KG, Germany) for ammonium and nitrate. Water in the aquaria was changed when values exceeded 0.4 mg/l and 0.2 mg/l of ammonium and nitrate, respectively.

### Fluorometric analyses

Several physiological parameters and free radical formation were assessed by live-imaging techniques, applying specific dyes and *ex-vivo* visualization using a Leica TCS SP5II confocal microscope (Leica Microsystems CMS GmbH, Wetzlar, Germany) equipped with a multiphoton laser (MaiTai-DeepSee, Spectra-Physics, Newport Corp.).

For each mussel analyzed, three freshly excised demibranch pieces (Fig. 1) were transferred to sterile medium composed of seawater filtered over a 0.2 μm Whatman filter and supplemented with 15 mM Na-HEPES (NH-FSW) and 0.5 mM glucose. These *ex-vivo* tissue samples were incubated with different dyes, the chemical reaction mechanisms, concentrations and conditions of visualization are summarized in Table 1. In order to confirm staining patterns, between three and five animals were independently stained with each dye (see Table 1). Another three animals were used as controls to adjust the threshold for fluorophore staining and suppress the autofluorescence of the samples under the analytical conditions for each of the dyes. The formation of superoxide anion (O_2_^•-^) was assessed by incubating samples in a NH-FSW medium supplemented with 10 μM DHE (Dihydroethidium, Molecular Probes D-23107, 0.5 mM stock solution in DMSO) whereas a number of other ROS species, e.g. H_2_O_2_, HOO^•^ and ONOO^−^ but not O_2_^•-^, were assessed using 20 μM C-H_2_DFFDA (Molecular Probes C-13293, 2 mM stock solution in Ethanol) in NH-FSW. Both stainings lasted 30 min. Both of these dyes were previously tested and successfully used in a previous study by Rivera-Ingraham et al. [45], in which the effect of anoxia and anoxia-reoxygenation on ROS production was tested in *M. edulis* gill tissues. In these experiments the fluorescence was suppressed in anoxia and massively increased over normoxic control during reoxygenation, indicating the expected ROS burst (see Fig. 3 of Rivera-Ingraham et al. [45]). Nitric oxide (NO) formation was visualized through DAF-2DA staining for 30 min (Sigma D224, 5 mM in DMSO) using a final concentration of 20 μM in NH-FSW.

**Table 1:** Analysis conditions for each of the dyes used during the study

					Excitation		Emission		
Dye	Mechanism of function	N	Conc. (μM)	Incub. time (min)	λ_1_ (nm)	λ_2_ (nm)	PMT1 (nm)	PMT2 (nm)	Calculation
Ageladine-A (in DMSO)	Probe which exists as a nearly uncharged monomer under pH of 8.1–8.6. After crossing cellular membranes, becomes charged in the cytosol and acidic compartments of cells.	3	30	90	MP 760	-	420-500	-	Average intensity
C-H_2_DFFDA (in Ethanol)	Non-fluorescent molecule which is converted to a green-fluorescent form (DCF) when the acetate groups are removed by intracellular esterases and oxidation occurs in the cell.	3	20	30	488	-	510-550	-	Average Intensity
DAF-2DA (in DMSO)	DAF-2 is formed by intracellular hydrolization of its ester bonds by esterases. It remains essentially non-fluorescent until it reacts with nitrosonium cation (forming the fluorescent DAF-2 T) and such fluorescence increases in a NO-dependent manner.	5	20	30	488	-	505-525	-	Average Intensity
Dihydroethidium (DHE) (in DMSO)	Regularly shows a blue emission when excited with a 355 nm laser. When oxidized to 2-dihydroxiethidium (2-OH-E^+^) by the presence of O_2_^−^, it intercalates with the DNA and shows a red emission when excited with an argon laser.	5	10	30	MP 710	488	400–440	620–660	Ratio PMT2/PMT1
JC-1 and JC-10 (in DMSO)	Green fluorescent probe which exists as a monomer at low Δ_ψm_. With high Δ_ψm_ values, JC-1 and JC-10 aggregates and shows a red fluorescence.	3	10	60	488	488	500–550	560–600	Ratio PMT1/PMT2
Lysotracker Red DND-99 (in DMSO)	Cell permeable fluorophore, which through protonation, concentrates on the membranes of spherical acidic organelles.	3	0.07	5	577	-	580–620	-	Average intensity
MitoTracker Deep Red 633 (in DMSO)	Molecule which becomes evidently fluorescent once accumulates in the lipid environment of the mitochondria.	4	1	60	633	-	640–680	-	Average intensity
Syto-13 (in DMSO)	Dye which upon binding to nucleic acid exhibits a green fluorescence.	3	10	60	488	-	500–520	-	Average Intensity

All MP analyses conducted with 2% pulsing laser power of around 2 W

MP=Multiphoton laser

Gill tissues were additionally stained with SYTO-13 (Molecular Probes S7575, 5 mM in DMSO) and Mitotracker (MTK) Deep Red 633 (Molecular Probes M-22426, 1 mM stock solution in DMSO) in order to locate the nuclei and observe the variations in mitochondrial density, respectively. Staining with the pH-sensitive dye ageladine-a [59] (Marnas Biochemicals GmbH) allowed observations of pH gradients along filaments. JC-1 (Molecular Probes D-23107 in DMSO) or JC-10 (Enzo Life Sciences ENZ-52305, 1 mM stock solution in DMSO) staining were used to observe the differences in mitochondrial membrane potential (Δψm) along the filaments of *M. edulis* gills. During the course of the experiments we changed from using JC-1 to JC-10 because of its better solubility in aqueous media. The validity of JC-1 staining, or of the derivative JC-10, for the detection of changes of Δψm has previously been documented for invertebrate models (e.g. [60]), including bivalve cells (e.g. [61, 62]). In order to prove the validity of the JC-1 or JC-10 signals in *Mytilus edulis* cells, we performed time series measurements using gill pieces stained with JC-10 to which we added 5 mM ADP to activated mitochondrial energetic coupling, expected to result in reduced Δψm.

Dyes were used individually for quantification and analysis to avoid possible interference among them. When possible, and only for further verification of the results, samples were stained with a combination of dyes, e.g., double staining was performed using Syto-13 and MTK Deep Red 633. In order to avoid photo-bleaching, a short period (<5 s) of lower resolution (512 × 512 pixel) live scanning was applied for focal adjustments and afterwards only one single scan (1024 × 1024 pixel) was run for each individual sample. Image analysis and fluorescence quantification was carried out as detailed in Rivera-Ingraham et al. [45] and with some modifications: briefly, a minimum of 5 pictures were taken across the surface of the dissected tissue. For each of the pictures taken, a total of 5 transects (or regions of interest, ROI) were plotted perpendicularly to the longitudinal axis of the gill filament and on the areas of the highest fluorescence intensity. Fluorescence intensity across the length of ROIs was quantified using Leica LAS AF Lite software (Leica Microsystems CMS GmbH 2011).

### Enzymatic antioxidant activity and oxidative damage

For a total of 9 specimens, gill tissues were dissected and the ventral bends were separated from the rest of the tissue. Both gill sections were weighed and stored at −80 °C until further analysis. All samples were homogenized in a 30 mM KPi-120 mM KCl buffer (pH 7.4) supplemented with a cocktail of protease inhibitors: phenylmethylsulfonyl fluoride (PMSF) (20 mg/ml in isopropanol), leupeptin (0.01 g/ml), pepstatin A (0.9 mg/ml in 90/10 ethanol/acetic acid) and aprotinin (0.01 g/ml). CAT activity was determined as the decomposition of a 0.3 M H_2_O_2_ solution (modified after [63]) and SOD activity was measured using the cytochrome oxidase assay after Livingstone et al. [64]. Oxidative damage was assessed as PC content using the OxiSelect Protein Carbonyl ELISA Kit (Cell Biolabs Inc., San Diego, CA) according to the manufacturer’s instructions. All values were related to protein content measured by the method originally described by Bradford [65].

### Statistical analyses

Two gill areas were compared in the study (Fig. 1): the ventral bend area (SA1) and the filamental part of the gill (SA2). For filaments, and only in the fluorometric testings, two different regions were independently analyzed: the epithelial cells and the blood sinus. For further fluorometric analyses, epithelial cells were subdivided in two equally sized regions at higher image resolution (outer and inner regions, in all cases excluding the blood sinus). All data are expressed as mean ± s.e.m. All data sets were tested for normality and homocedasticity through a Kolmogorov-Smirnov and Levene’s Tests, respectively. When data complied with both of these assumptions, ANOVA tests were conducted. Otherwise, a Kruskal-Wallis test was carried out. Statistical analyses were performed using SPSS 15.0 (SPSS Inc., Chicago, IL, USA).

## Abbreviations

CAT: catalase
MTK: mitotracker
NH-FSW: Na-HEPES-supplemented filtered seawater
NO: nitric oxide
PC: protein carbonyls
pO_2_: Oxygen partial pressure
RNS: reactive nitrogen species
ROS: reactive oxygen species
SMG: subepithelial mucus gland
SOD: superoxide dismutase
ΔΨm: mitochondrial membrane potential

## References

[1] Reid MB (2001). Redox modulation of skeletal muscle contraction: what we know and what we don’t. J Appl Physiol.

[2] Dröge W (2002). Free radicals in the physiological control of cell function. Physiol Rev.

[3] Turrens JF (2003). Mitochondrial formation of reactive oxygen species. J Physiol.

[4] Nikinmaa M, Gassmann M, Bogdanova A, Abele D, Vázquez-Media JP, Zenteno-Savín T (2012). Oxygen sensing: the role of reactive oxygen species. Oxidative stress in aquatic ecosystems.

[5] Welker AF, Moreira DC, Campos EG, Hermes-Lima M (2013). Role of redox metabolism for adaptation of aquatic animals to drastic changes in oxygen availability. Comp Biochem Physiol A Physiol.

[6] Gensch E, Gallup M, Sucher A, Li D, Gebremichael A, Lemjabbar H (2004). Tobacco smoke control of mucin production in lung cells requires oxygen radicals AP-1 and JNK. J Biol Chem.

[7] Heise K, Puntarulo S, Pörtner HO, Abele D (2003). Production of reactive oxygen species by isolated mitochondria of the Antarctic bivalve *Laternula elliptica* (King and Broderip) under heat stress. Comp Biochem Phys.

[8] Philipp E, Pörtner H-O, Abele D (2005). Mitochondrial ageing of a polar and a temperate mud clam. Mech Ageing Dev.

[9] Donaghy L, Hong H-K, Lambert C, Park H-S, Shim WJ, Choi K-S (2010). First characterisation of the populations and immune-related activities of hemocytes from two edible gastropod species, the disk abalone, *Haliotis discus discus* and the spiny top shell, *Turbo cornutus*. Fish & shellfish immunology.

[10] Donaghy L, Kim B-K, Hong H-K, Park H-S, Choi K-S (2009). Flow cytometry studies on the populations and immune parameters of the hemocytes of the Suminoe oyster, *Crassostrea ariakensis*. Fish & shellfish immunology.

[11] Viarengo A, Burlando B, Cavaletto M, Marchi B, Ponzano E, Blasco J (1999). Role of metallothionein against oxidative stress in the mussel *Mytilus galloprovincialis*. Am J Physiol Regul Integr Comp Physiol.

[12] Ferreira-Cravo M, Reinhardt Piedras F, Barros Moraes T, Ribas Ferreira JL, de Freitas DP S, Dornelles Machado M (2007). Antioxidant responses and reactive oxygen species generation in different body regions of the estuarine polychaeta *Laeonereis acuta* (Nereididae). Chemosphere.

[13] Brady NR, Elmore SP, van Beek JJHGM, Krab K, Courtoy PJ, Hue L (2004). Coordinated behaviour of mitochondria in both space and time: a reactive oxygen species-activated wave of mitochondrial depolarization. Biophys J.

[14] Kristiansen KA, Jensen PK, Møller IM, Schulz A (2009). Monitoring reactive oxygen species formation and localisation in living cells by use of the fluorescent probe CM-H2DCFDA and confocal laser microscopy. Physiol Plant.

[15] Van Winkle WJ (1972). Ciliary activity and oxygen consumption of excised bivalve gill tissue. Comp Biochem Physiol A Mol Integr Physiol.

[16] Ward JE (1996). Biodynamics of supension-feeding in adult bivalve molluscs: particle capture, processing, and fate. Invertebr Biol.

[17] Jørgensen C (1996). Bivalve filter feeding revisited. Mar Ecol Prog Ser.

[18] Hodgson AN, Fielden LJ (1984). The structure and distribution of peripheral ciliated receptors in the bivalve molluscs Donax serra and D sordidus. J Molluscan Stud.

[19] Beninger PG, Dufour SC, Harper EM, Taylor JD, Crame JA (2000). Evolutionary trajectories of a redundant feature: lessons from bivalve gill abfrontal cilia and mucocyte distributions. The Evolutionary Biology of the Bivalvia.

[20] Riisgård HU, Larsen PS, Nielsen NF (1996). Particle capture in the mussel *Mytilus edulis:* the role of latero-frontal cirri. Mar Biol.

[21] Beninger PG, St-Jean S, Poussart Y, Ward JE (1993). Gill function and mucocyte distribution in *Placopecten magellanicus* and *Mytilus edulis* (Mollusca: Bivalvia): the role of mucus in particle transport. Mar Ecol Prog Ser.

[22] Beninger PG, Lynn JW, Dietz TH, Silverman H (1997). Mucociliary transport in living tissue: the two-layer model confirmed in the mussel *Mytilus edulis* L. Biol Bull.

[23] Gómez-Mendikute A, Elizondo M, Venier P, Cajaraville MP (2005). Characterization of mussel gill cells in vivo and in vitro. Cell Tissue Res.

[24] Bickmeyer U (2012). The alkaloid Ageladine A, originally isolated from marine sponges, used for pH-sensitive imaging of transparent marine animals. Marine Drugs.

[25] Rivera-Ingraham GA, Bickmeyer U, Abele D (2013). The physiological response of the marine platyhelminth *Macrostomum lignano* to different environmental oxygen concentrations. J Exp Biol.

[26] Kratzing JE (1982). Regional variation in respiratory epithelium of the nasal cavity of the bandicoot (*Isoodon macrourus*). J Anat.

[27] Duncan JR, Ramsey FK (1965). Fine structural changes in the porcine nasal ciliated epithelial cell produced by *Bordetella bronchiseptica* rhinitis. Am J Pathol.

[28] Domouhtsidou GP, Dimitriadis VK (2004). Lysosomal, tissue and cellular alterations in the gills, palps and intenstine of the mussel *Mytilus galloprovincialis,* in relation to pollution. Mar Biol.

[29] Kádár E, Lowe DM, Solé M, Fisher AS, Jha AN, Readman JW (2010). Uptake and biological responses to nano-Fe versus soluble FeCl_3_ in excised mussel gills. Anal Bioanal Chem.

[30] Kasuya K, Miyoshi M (2001). Fine structure of cilia and basal bodies, with reference to the mouse trachea. Medical Bulletin of Fukuoka University.

[31] Di Lisa F, Blank P, Colonna R, Gambassi G, Silverman H, Stern M (1995). Mitochondrial membrane potential in single living adult rat cardiac myocytes exposed to anoxia or metabolic inhibition. J Physiol.

[32] Smiley ST, Reers M, Mottola-Hartshorn C, Lin M, Chen A, Smith TW (1991). Intracellular heterogeneity in mitochondrial membrane potentials revealed by a J-aggregate-forming lipophilic cation JC-1. Proc Natl Acad Sci U S A.

[33] Collins TJ, Berridge MJ, Lipp P, Bootman MD (2002). Mitochondria are morphologically and functionally heterogeneous within cells. The EMBO Journal.

[34] Reers M, Smiley ST, Mottola-Hartshorn C, Chen A, Lin M, Chen LB (1995). Mitochondrial membrane potential monitored by JC-1 dye. Methods Enzymol.

[35] Skulachev VP (2001). Mitochondrial filaments and clusters as intracellular power-transmitting cables. Trends Biochem Sci.

[36] Brown GC (1999). Nitric oxide and mitochondrial respiration. Biochim Biophys Acta.

[37] Buchwalow IB, Podzuweit T, Bocker W, Samoilova VE, Thomas S, Wellner M (2002). Vascular smooth muscle and nitric oxide synthase. FASEB J.

[38] Marechal G, Gailly P (1999). Effects of nitric oxide on the contraction of skeletal muscle. Cell Mol Life Sci.

[39] Reid MB (1998). Role of nitric oxide in skeletal muscle: synthesis, distribution and functional importance. Acta Physiol Scand.

[40] Brady AJB, Warren JB, Poole-Wilson PA, Williams TJ, Harding SE (1993). Nitric oxide attenuates cardiac myocyte contraction. Am J Physiol.

[41] Schipp R, Gabauer M (1999). Nitric oxide: a vasodilatadory mediator in cephalic aorta of *Sepia officinalis* (L.) (Cephalopoda). Invertebr Neurosci.

[42] Fang FC (1997). Mechanisms of nitric oxide-related antimicrobial activity. J Clin Investig.

[43] Palumbo A (2005). Nitric oxide in marine invertebrates: a comparative perspective. Comp Biochem Physiol A Mol Integr Physiol.

[44] Stefano GB, Ottaviani E (2002). The biochemical substrate of nitric oxide signaling is present in primitive non-cognitive organisms. Brain Res.

[45] Rivera-Ingraham GA, Rocchetta I, Meyer S, Abele D (2013). Oxygen radical formation in anoxic transgression and hypoxia-reoxygenation: foe or phantom? Experiments with an anoxia tolerant bivalve. Mar Environ Res.

[46] Abele-Oeschger D, Oeschger R (1995). Hypoxia induced autoxidation of haemoglobin in the benthic invertebrates *Arenicola marina* (Polychaeta) and *Astarte borealis* (Bivalvia): possible effect of hydrogen sulphide. J Exp Mar Biol Ecol.

[47] Nakamura M, Mori K, Inooka S, Nomura T (1985). *In vitro* production of hydrogen peroxide by the amoebocytes of the scallop, *Patinopecten yessoensis* (Jay). Dev Comp Immunol.

[48] Le Gall G, Bachère E, Mialhe E (1991). Chemiluminiscence analysis of the activity of *Pecten maximus* hemocytes stimulated with Zymosan and host-specific Rickettsiales-like organisms. Disease of Aquatic Organisms.

[49] Larson KG, Roberson BS, Hetrick FM (1989). Effect of environmental pollutants on the chemiluminiscence of hemocytes from the American oyster *Crassostrea virginica*. Disease of Aquatic Organisms.

[50] Bachère E, Hervio D, Mialhe E (1991). Luminol-dependent chemiluminiscence by hemocytes of two marine bivalves, *Ostrea edulis* and *Crassostrea gigas*. Disease of Aquatic Organisms.

[51] Pipe RK (1992). Generation of reactive oxygen metabolites by the haemocytes of the mussel *Mytilus edulis*. Dev Comp Immunol.

[52] Winston GW, Moore MN, Kirchin MA, Soverchia C (1996). Production of reactive oxygen species by hemocytes from the marine mussel, *Mytilus edulis*: lysosomal localization and effect of xenobiotics. Comp Biochem Physiol C Toxicol Pharmacol.

[53] Kalyanaraman B, Darley-Usmar VM, Davies KJA, Dennery PA, Forman HJ, Grisham MB (2012). Measuring reactive oxygen and nitrogen species with fluorescent probes: challenges and limitations. Free Radic Biol Med.

[54] Prakash P, Kumar GP, Laloraya M, Hemnani T, Parihar MS (1998). Superoxide anion radical generation as a temperature stress response in the gills of freshwater catfish *Heteropneustes fossilis:* role in mucus exudation under elevated temperature. Comp Biochem Physiol C.

[55] Segal AW (2008). The function of the NADPH oxidase of phagocytes and its relationship to other NOXs in plants, invertebrates, and mammals. Int J Cell Biol.

[56] Proctor P, Kirkpatrick D, Mc-Ginnes J (1977). A superoxide-producing system in the conjuctival mucus thread. Investig Ophthalmol Vis Sci.

[57] Teshima S, Rokutan K, Nikawa T, Kishi K (1998). Guinea pig gastric mucosal cells produce abundant superoxide anion through an NADPH oxidase-like system. Gastroenterology.

[58] Strahl J, Abele D (2010). Cell turnover in tissues of the long-lived ocean quahog *Arctica islandica* and the short-lived scallop *Aequipecten opercularis*. Mar Biol.

[59] Bickmeyer U, Grube A, Klings K-W, Köck M (2008). Ageladine A, a pyrrole–imidazole alkaloid from marine sponges, is a pH sensitive membrane permeable dye. Biochem Biophys Res Commun.

[60] Julian D, April KL, Patel S, Stein JR, Wohlgemuth S (2005). Mitochondrial depolarization following hydrogen sulfide exposure in erythrocytes from a sulfide-tolerant marine invertebrate. J Exp Biol.

[61] Haberkorn H, Lambert C, Le Goïc N, Moal J, Suquet M, Guéguen M (2010). Effects of *Alexandrium minutum* exposure on nutrition-related processes and reproductive output in oysters *Crassostrea gigas*. Harmful algae.

[62] Donaghy L, Kraffe E, Le Goïc N, Lambert C, Volety AK, Soudant P (2012). Reactive oxygen species in unstimulated hemocytes of the Pacific oyster *Crassotrea gigas*: a mitochondrial involvement. PLoS One.

[63] Aebi H (1984). Catalase in vitro. Methods Enzymol.

[64] Livingstone DR, Lips F, Garcia Martinez P, Pipe RK (1992). Antioxidant enzymes in the digestive gland of the common mussel *Mytilus edulis*. Mar Biol.

[65] Bradford MM (1976). A rapid and sensitive method for the quantitation of microgram quantities of protein utilizing the principle of protein-Dye binding. AnalBiochem.

